# Spatiotemporal Mapping of Biomechanical Stress Predicts Region-Specific Retinal Injury in a Murine Model of Blunt Ocular Trauma

**DOI:** 10.3390/bioengineering13040431

**Published:** 2026-04-07

**Authors:** Jianing Wang, Ji An Lee, Yingnan Zhai, Kourosh Shahraki, Pengfei Dong, Donny W. Suh, Linxia Gu

**Affiliations:** 1 Department of Biomedical Engineering and Science, Florida Institute of Technology, Melbourne, FL 32901, USA; jianing2016@my.fit.edu (J.W.); yzhai2016@my.fit.edu (Y.Z.); pdong@fit.edu (P.D.); 2 School of Medicine, Creighton University, Phoenix, AZ 85012, USA; jianlee@creighton.edu; 3 Department of Ophthalmology and Visual Sciences, Gavin Herbert Eye Institute, University of California, Irvine, CA 92617, USA; kourosh.shahyar@gmail.com

**Keywords:** blunt traumatic eye injury, retinal injury, finite element analysis

## Abstract

Retinal detachments following blunt ocular trauma are challenging to predict due to the complex and transient biomechanical responses of the globe. This study combines an in vitro weight-drop experiment and finite element analysis (FEA) to evaluate the mechanical pathways leading to traumatic retinal detachment and to predict the spatial likelihood of injury. In the in vitro model, a cylindrical weight was impacted onto freshly enucleated mouse eyes (16 weeks old) supported on a rigid metal plate. Following impact, the eyes were sectioned and stained using hematoxylin and eosin (H&E) for histological assessment. A finite element model of a mouse eye, including the cornea, sclera, lens, zonule, vitreous body, aqueous humor, and retina, was reconstructed from the histological section and used to simulate the whole sequence of compression and rebound following the blunt impact. The simulation demonstrated that the lens retained a high momentum. It generated an alternating compressive (up to −6.57 × 10^−3^ MPa) and tensile (up to 1.62 × 10^−3^ MPa) radial stress at the posterior pole and sustained compressive stress at the peripheral region (up to −3.12 × 10^−3^ MPa) and tensile-compressive stress variation at the equatorial region of the retina. In addition, the regions experiencing tensile stress overlapped with the region exhibiting retinal detachment in the in vitro experiment. These findings highlight the spatiotemporal mapping of biomechanical stress to predict traumatic retinal detachment following blunt impact and provide an understanding of early biomechanical response following ocular trauma.

## 1. Introduction

Ocular trauma is one of the leading causes of visual impairment and blindness worldwide. Epidemiological data estimate that approximately 2.4 million cases of ocular trauma occur each year in the United States alone [1], with common causes including foreign bodies, mechanical force, and falls [2]. Unintentional traumatic eye injuries from objects and machinery are the greatest contributors to ocular trauma in children, accounting for approximately 30.1% of cases [3]. Blunt ocular trauma, in particular, comprises about 37.8% of all traumatic eye injuries and poses a significant risk for unilateral vision loss and permanent blindness.

Blunt forces on the eye can cause rapid distortion of the globe, which leads to damage to multiple regions of the eye, including anterior, lens, and posterior segments. Injury on the anterior segment is usually due to direct physical or chemical exposure, causing corneal abrasions, hyphemia, lens dislocation and globe rupture. Posterior segment injury is more often associated with indirect mechanical forces transmitted through the globe, such as deformation, pressure waves, traction, or rebound effects, rather than direct exposure, but is often linked to more severe and permanent vision loss [4], such as commotio retinae, vitreous hemorrhage, retinal detachment, choroidal rupture, retinal hemorrhage and tear [5]. The pathogenesis of traumatic retinal detachment (TRD) is attributed to acute, transient vitreoretinal traction and mechanical disruption of the retinal layers from the underlying retinal pigment epithelium (RPE). Following blunt ocular trauma, rapid globe deformation produces retinal tears and dialysis, particularly at sites of vitreoretinal adhesion such as the vitreous base. Once a retinal break forms, liquefied vitreous bodies can pass into the subretinal space and separate the neurosensory retina from the RPE, leading to progressive detachment [6,7]. Predicting the specific location and likelihood of retinal detachment following blunt impact remains a formidable clinical challenge. The difficulty stems from the complex, dynamic, and rapid biomechanical deformation of the globe, where forces are transmitted and dissipated through a composite structure of fluids and viscoelastic tissues.

Blunt ocular trauma has been extensively studied using enucleated animal eyes, such as those from rodents and mammals, due to their anatomical and cellular resemblance to human eyes, as well as their availability and experimental reproducibility in laboratory settings [8]. Some prior studies have simulated ocular injury using high-velocity projectiles, such as modified air guns, to model ocular trauma in porcine eyes [9]. Similarly, the weight-drop model has been employed as a controlled and repeatable method to induce ocular trauma and assess the associated structural and tissue responses [10].

Despite the advantages of ex vivo experimentation, the transient structural behavior during blunt ocular trauma experiments is hard to quantify [11]. While these models enable histological correlation, they are inherently limited in capturing the full-field, time-resolved mechanical stress states within the eye during the milliseconds of impact. Therefore, finite element analysis (FEA) has been widely adopted as a computational tool to investigate the biomechanical mechanisms of ocular trauma [12]. By constructing a model eye in silico that integrates realistic anatomical geometry and tissue properties, FEA enables detailed prediction of stress distribution, tissue deformation, and potential injury sites under dynamic traumatic conditions [13]. This modeling framework has been successfully applied in the assessment of sports-related ocular injuries and abusive head trauma [14,15,16]. For example, Rangarajan et al. developed FEA to investigate the ocular injury mechanism associated with abusive head trauma [17], and Grey et al. used FEA to simulate the different parameters of paintball impacts on the eye [18]. Recent studies have highlighted the importance of combining FEA with experimental support to improve model accuracy. Chen et al. integrated FEA simulation with an in vitro blunt-impact test on porcine eyes, achieving close agreement in predicting retinal detachment sites under different impact conditions [19]. Our study employs a more detailed murine eye model, which can identify stress patterns surrounding the retina associated with retinal detachment. The murine eye offers a well-characterized system with significant genetic and anatomical homology to humans, facilitating precise histological comparison.

In this study, we aim to advance our understanding of vitreoretinal traction and retinal injury during blunt ocular trauma by mapping the spatiotemporal distribution of biomechanical stress in the retina. Tensile stress distribution in the retina from FEA was compared with sites of retinal separation from histological analysis following in vitro weight-drop experiments on enucleated murine eye models. By integrating computational simulation with in vitro experiments, this study provides a novel framework for investigating the early biomechanical response following ocular trauma that may cause retinal detachment.

## 2. Materials and Methods

### 2.1. Computational Simulation of Weight Drop

**Figure 1:**
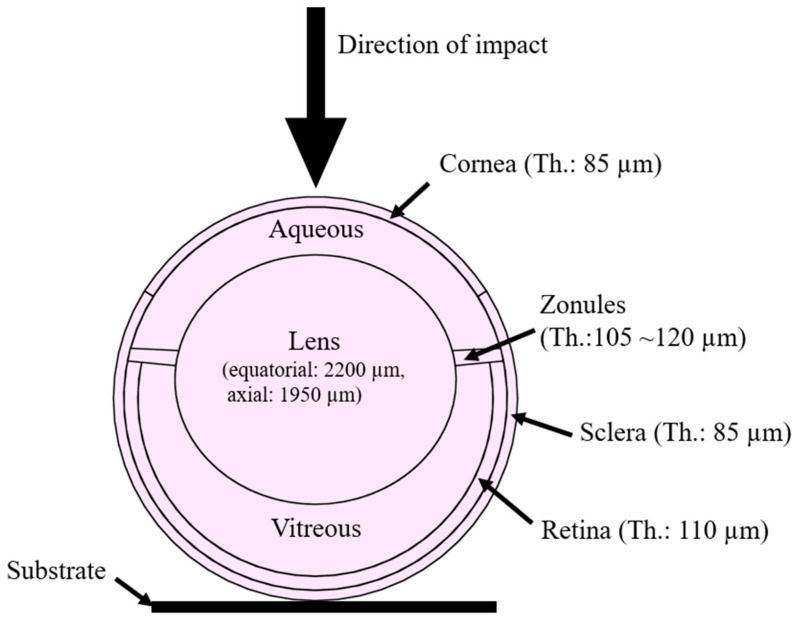
FEA murine eye model in cross-section view. The model is reconstructed from histological sectioning. Th.: thickness.

An idealized model of a mouse eye was reconstructed using Fusion 360 (Autodesk, Inc., San Rafael, CA, USA) based on the histological cross-section data from the literature [20]. This includes the cornea, aqueous humor, zonular fibers, lens, vitreous body, sclera, and retina (Figure 1). The eyeball diameter is 3.10 mm with cornea, scleral and retinal thicknesses of 80 µm, 80 µm and 110 µm, respectively. The lens is a near spherical shape with an axial thickness of 1950 µm and an equatorial diameter of 2200 µm. The zonule thickness was 120 µm at the lens side and 105 µm at the scleral side. The impactor was modeled as a rigid cylinder with a diameter of 6 mm. The setup is consistent with an in vitro weight-drop test. The eye was positioned with the anterior cornea facing upward and supported by a rigid flat substrate under the posterior sclera. The impactor was assigned an initial velocity of 1.00 m/s, equivalent to a speed reached by a free fall from 60 mm above the cornea.

The biomechanical properties of each tissue were obtained from the literature and are summarized in Table 1. The cornea, sclera, and lens were modeled as hyperelastic materials, and the zonules were modeled as linear elastic materials, as shown in Table 1. Aqueous humor and vitreous bodies were defined as Eulerian domains and assigned with an equation of state material model. The eye model was discretized using tetrahedral elements (SOLID 187). Based on the mesh-convergence studies, the final mesh was selected when the successive mesh refinement produced a small change in the maximum von Mises stress of the model (8.5% in this study). The mesh numbers of all components are listed in Table 1. Topology sharing was applied at the cornea–sclera, zonule–sclera, lens–zonule, zonule–retina, and retina–sclera interfaces to enable node sharing between adjacent tissues. The frictionless contact was defined between the impactor and the cornea and between the sclera and the substrate.

The simulation was performed using the explicit dynamic solver in ANSYS 2024R1 (ANSYS Inc., Canonsburg, PA, USA), with a total simulation time of 5 ms, which captured the entire duration of contact and rebound between the impactor and the eyeball. Normal stress components along x, y and z axes on the retinal surface were extracted in Cartesian coordinates at the geometric center of the retinal layer. These values were then transferred from Cartesian to spherical coordinates to obtain the normal stress components (σ_r_,σ_θ_,σ_φ_), where r represents the radial axis, θ the polar angle, and φ the azimuthal angle, using the following equations:

(1) $$r=\sqrt{x^{2}+y^{2}+z^{2}}$$

(2) $$\theta =\cos\left(\frac{z}{\sqrt{x^{2}+y^{2}+z^{2}}}\right)$$

(3) $$\varphi =\tan\left(\frac{y}{z}\right)$$

and

(4) $$\left[\begin{array}{c} \sigma_{r}\\ \sigma_{\theta }\\ \sigma_{\varphi } \end{array}\right]=\left[\begin{array}{ccc} \sin\theta \cos\varphi & \sin\theta \sin\varphi & \cos\theta \\ \cos\theta \cos\varphi & \cos\theta \sin\varphi & -\sin\theta \\ -\sin\varphi & \cos\varphi & 0 \end{array}\right]\left[\begin{array}{c} \begin{array}{c} \sigma_{x}\\ \sigma_{y}\\ \sigma_{z} \end{array} \end{array}\right]$$

Radial stress, σ_r_, in the retina was measured to analyze mechanical responses during impact. Because the eyeball model is asymmetric, this study focused on the spatial variation of σ_r_ with respect to the polar angle θ, which was measured as the distance from the posterior pole of the retina. For analysis, the retina was divided into three anatomical regions: the posterior pole, peripheral, and equatorial region. The stress distribution in the retina was analyzed at two distinct phases: the impact phase and the retraction phase, which differ according to the lens displacement. The maximum radial stress in these regions was extracted to further analyze the tensile stress behavior at different time points.

### 2.2. In Vitro Weight-Drop Test on Mouse Eye and Histological Assessment

**Figure 2:**
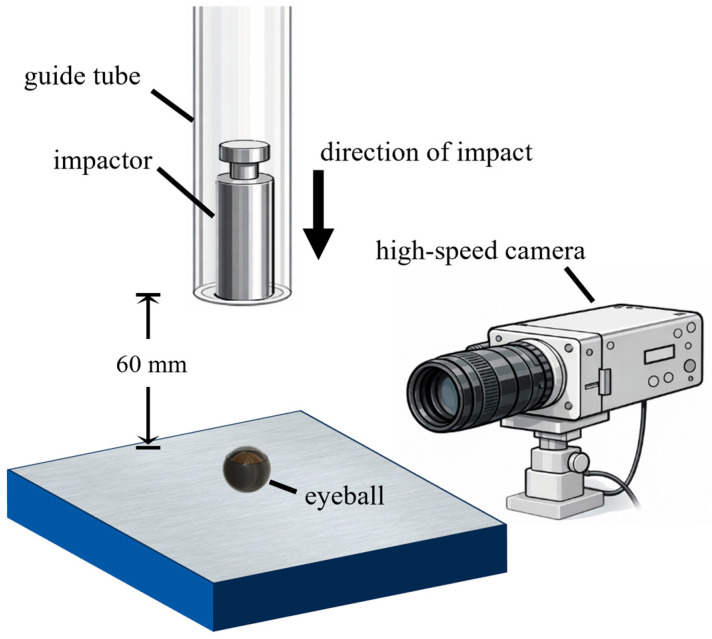
Schematic illustration of the weight-drop impact setup on mouse eye.

The weight-drop tests were conducted on freshly extracted eyes from 16-week-old mice. Animal care and the protocol were conducted following approval by the Florida Institute of Technology’s Institutional Animal Care and Use Committee (#2022-04, approval date 12 February 2025). The extracted eyes were inspected to ensure no visible globe damage occurred during the extraction process. For the weight-drop group, each eye was positioned with the cornea facing superiorly, and a cylindrical impactor, with a 5 g mass and a 6 mm diameter, was released from a height of 60 mm above the cornea. A guide tube was used to ensure alignment between the drop path and the center of the cornea. The entire test was recorded using the high-speed camera, FASTCAM Nova S6 (Photron, San Diego, CA, USA) to verify the impact velocity and impact location (Figure 2). The recordings were used to verify that the impactor struck the cornea vertically. The eyes impacted off-center or on the side were excluded from further analysis. Ultimately, three eyes were used for the in vitro weight-drop test, and three eyes were in the control group. Following the impact test, the eyeball was immediately fixed in 4% paraformaldehyde for 2 h then cryosectioned on the horizontal plane of the eye [21,25]. The middle slice closest to the optic nerves was selected for hematoxylin and eosin (H&E) staining. Histological analysis of the retinal layers assessed structural integrity and evidence of retinal detachment. For the control group, eyes were not subjected to impact testing but were collected and processed under the same fixation and histological procedures for comparison.

## 3. Results

### 3.1. Stress Distribution in Retina

**Figure 3:**
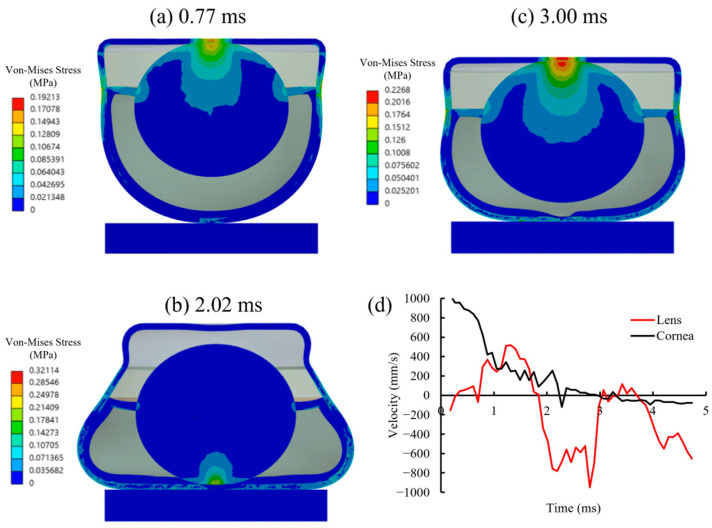
Blunt trauma to the murine eyeball. (**a**) Von Mises stress distribution at 0.77 ms. The stress concentrated on the central cornea and sclera–zonules junction; (**b**) von Mises stress distribution at 2.02 ms. The momentum was transferred into the lens that contacted the retina. The stress concentrated on the posterior pole and peripheral region of the retina; (**c**) von Mises stress distribution at 3.00 ms. The lens rebounded and contacted the cornea again. The stress concentrated on the central cornea, sclera–zonules junction and retina; (**d**) variation in velocity of lens and cornea.

The impact normal to the cornea induced sufficient momentum into the eyeball. The stress concentration was observed in the central cornea and lens at 0.77 ms (Figure 3a). The lens accelerated and stretched the zonules and ultimately generated a stress concentration at the joint to the sclera and equatorial retina. The lens maintained a relatively high velocity (Figure 3d) until it hit the retina at 2.02 ms, which generated stress concentration at the posterior pole (Figure 3b). Meanwhile, the posterior chamber bulged, and stress was concentrated at the peripheral region, resulting not only from the lens momentum but also from compression of the vitreous body. The lens rebounded and re-contacted the cornea at 3.00 ms (Figure 3c). The posterior bulging retina was slightly subsided, but the stress remained concentrated in the equatorial region. Figure 3d represents the momentum transferred between the cornea and the lens. The slightly decreased velocity reflects the damping effect of the zonules and the aqueous humor. A similar phenomenon occurred in the posterior chamber, where the vitreous body maintained its bulged shape and likely transmitted momentum to the retinal tissue, increasing the risk of local detachment due to pressure fluctuations.

**Figure 4:**
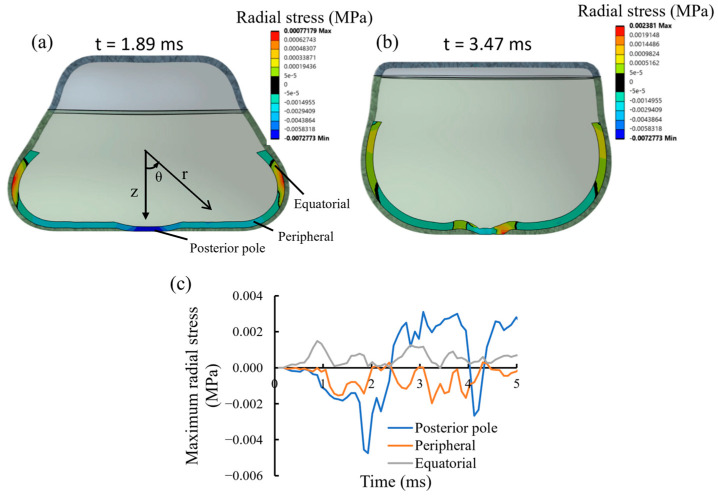
Radial stress in retina along the radial axis, **r**, at (**a**) 1.89 ms and (**b**) 3.47 ms during the impact and retraction phases; the black bands represent the boundary between compressive (negative) and tensile (positive) stress of the retina; (**c**) the maximum radial stress in the posterior pole, peripheral, and equatorial vs. time.

The radial stress, σ_r_, distribution along the r-axis of the spherical coordinate system was modeled at two critical time points: during the impact phase (t = 1.89 ms) and during the retraction phase (t = 3.47 ms), with the origin located at the geometric center of the retina (Figure 4a,b). These points were chosen because, within each phase, the lens occupied the same relative position, which allowed a consistent comparison between the impact and retraction phases. The stress distribution was axisymmetric across the retinal surface, with positive stress representing a tensile force pulling the retina away from the sclera and negative stress representing a compressive force pressing the retina toward the sclera. During impact, the posterior pole, the peripheral, and the equatorial region were pressed toward the sclera with radial stresses up to about −7.27 × 10^−3^ MPa, −2.29 × 10^−3^ MPa, and 0.78 × 10^−3^ MPa at 1.89 ms (Figure 4a). In contrast, the posterior pole was pulled away from the sclera, forming tensile stress concentrations up to 1.45 × 10^−3^ MPa during the retraction phase. The peripheral and equatorial region remained pressed at −1.50 × 10^−3^ MPa and 0.98 × 10^−3^ MPa, respectively (Figure 4b). These findings suggest spatial discrepancies in retinal injury due to blunt trauma to the eye. In addition, temporal variation in stress distribution patterns across different regions may help explain the localized retinal detachment observed in post-impact histological analysis.

The maximum radial stress in the posterior pole, peripheral, and equatorial regions is shown in Figure 4c, where a negative value indicates that the entire corresponding region is under compressive stress, and a positive value indicates that at least some portions of the corresponding region are under tension. Over time, the posterior pole exhibited a greater variation in radial stress of the retina than the other two regions. The posterior pole sustained a peak compressive pressure of −4.75 × 10^−3^ MPa during the impact phase, which was over double that observed in the peripheral region (−2.08 × 10^−3^ MPa). During the retraction phase, the posterior pole began to pull away from the sclera, with a tensile stress in the retina reaching up to 3.11 × 10^−3^ MPa, followed by fluctuations of radial stress that pressed the retina once again. In contrast, the equatorial region exhibited a tensile-compressive stress variation but with relatively low values throughout both phases compared to peak stresses in the posterior pole.

### 3.2. Histological Analysis of Retinal Damage

**Figure 5:**
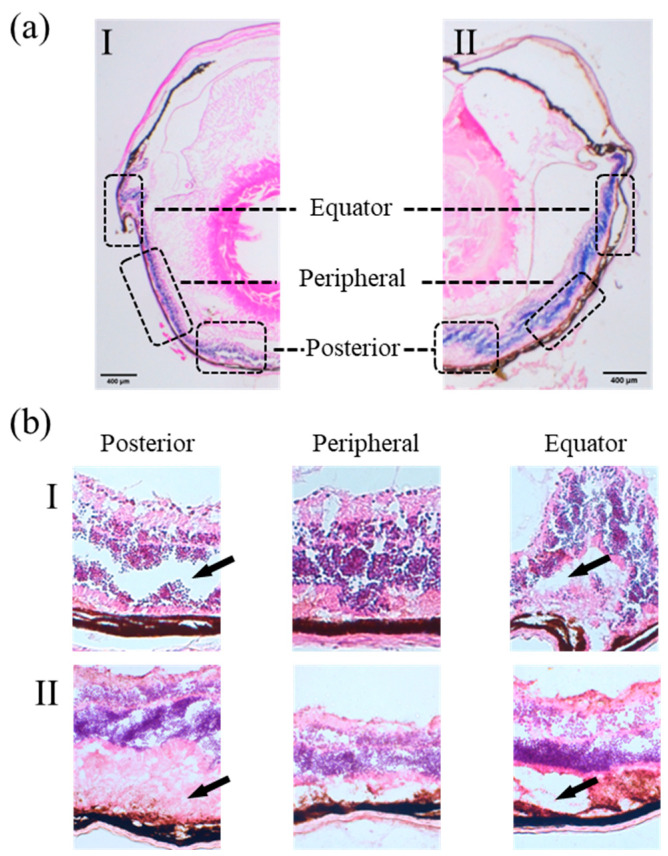
(**a**) Representative histological cross-sections of two mouse eyeballs (I and II) following weight-drop impact. Dashed boxes indicate regions of interest at the posterior pole, the peripheral region, and the equatorial region. (**b**) Localized views of the corresponding regions for each eyeball. Black arrows mark the sites of retinal detachment or stretching.

Histological examination of retina following weight-drop impact revealed region-specific patterns of structural disruption. Representative histological cross-sections of eyeballs following weight-drop impact are shown in Figure 5. At the posterior pole, distinct disruption was observed in all three examined eyes (3/3): a clear cleft in the outer nuclear layer (eyeball I) and stretching or elongation of the photoreceptor layer (eyeball II). At the equatorial region, similar signs of retinal dialysis were also observed in two of three eyes (2/3), where disruptions appeared in the photoreceptor layer (eyeball I & II) and the pigment epithelium (eyeball II). In contrast, the structure at the peripheral region is generally preserved with only one of three eyes (1/3) showing a small number of detachments of photoreceptor layer. The representative figures illustrated typical damage patterns observed across multiple samples. No consistent association was found between the globe regions and the specific layer affected. The histological comparison between the control and weight-drop eye is provided in Supplementary S1.

The histological observations and the corresponding FEA-derived stress patterns across retinal layers are summarized in Table 2. Although the histological variability in the extent of structural disruption and the specific retinal layer affected was observed across three eye samples, some common features were observed in the posterior pole, equatorial region, and peripheral region. In particular, the retinal detachment or stretching was more frequently observed in the posterior pole and equatorial region. These observations were compared with the tensile stress distribution in the retina from the simulation. An overlap was observed between regions experiencing tensile stress in the simulation and regions exhibiting retinoschisis and detachment on histology. Specifically, both the posterior pole and equatorial retina exhibited elevated tensile stresses that aligned with the observed structural disruptions in these regions. In contrast, the peripheral retina experienced compressive stress throughout the impact and exhibited minimal histological alterations. This correspondence between mechanical loading patterns and retinal tissue injury provided insight into how mechanical stress induces retinal detachment during blunt ocular trauma.

## 4. Discussion

This study provides a comprehensive biomechanical analysis of retinal injury following blunt ocular trauma by integrating finite element analysis (FEA) with a controlled in vitro murine model. Adhesion between the retinal layers and their supporting tissues is essential for maintaining retinal attachment. Blunt ocular impact can mechanically disrupt these interfaces, leading to partial or complete retinal detachment. Our results demonstrated that the posterior pole is at the greatest risk for vitreoretinal injury following blunt impact, followed by the equatorial region. The oscillatory stress resulting from alternating pulling and pushing forces applied to the retinal layer suggests a biomechanical mechanism underlying the retinal injuries. Previous work by Delori et al. described four sequential stages of globe deformation after blunt trauma: compression, decompression, overshooting, and oscillation [26]. Respectively, these phases involve a decrease in axial length, a return to baseline axial length, an extension beyond the baseline axial length, and fluctuations until stabilization to the original shape or the eyeball [27]. In our simulation, these phases are similarly portrayed. The posterior pole experienced the highest compressive radial stress during the initial compression phase, which subsequently shifted to tensile stress, where the force pulled the retinal layer from the sclera during the retraction phase. Although the compressive stress was higher than the tensile stress, the retina’s deformation was limited by the sclera’s much greater stiffness [28]. Where tensile force pulled the layer to the center of the vitreous body, the sole adhesive force of retinal pigment epithelium (RPE) counteracted its effect to detach and tear [13].

The FEA result and histological findings support that the posterior pole is prone to vitreoretinal injuries not only because of the largest blunt trauma force traveling anterior-posteriorly but also because of the greatest magnitude of rebound occurring during the overshooting and the oscillating phase. Disruption of the laminated RPE at the posterior pole in mouse histology aligns with the predicted stress distribution. A similar pattern has been reported in retrospective studies of blunt ocular trauma, in which the incidence of commotio retinae in the macular region was greater than that in the peripheral region following blunt ocular injury [29].

Together, these findings reinforce the concept that distinct anatomical zones of the retina are biomechanically predisposed to injury. Our data on the equatorial region, where moderate tensile stress from zonular traction corresponded with retinoschisis, underscores a second injury pathway. This mirrors clinical observations in abusive head trauma, where vitreoretinal traction during acceleration–deceleration forces leads to characteristic peripheral retinal hemorrhages and retinoschisis [30]. The splitting of retinal layers (retinoschisis) is a recognized precursor to full-thickness retinal detachment. Our model identifies the posterior pole and equatorial retina as biomechanical hotspots for TRD. This should guide clinicians to perform a meticulous examination of these regions, even in the absence of immediate symptoms, which are common in TRD. Enhanced imaging protocols can be developed based on these risk maps. For instance, wide-field optical coherence tomography (OCT) should be prioritized to detect subtle retinoschisis or outer layer tears in the equatorial region, while high-resolution macular OCT is crucial for assessing photoreceptor integrity and subclinical detachment at the posterior pole [31].

The momentum of the weight impact is rapidly transmitted into the lens through the cornea. As shown in the FEA results, the lens obtained a relatively high velocity and rapid posterior and anterior acceleration during both compression and rebound phases, which generated substantial stretch on the zonular fibers and the stress concentration on the limbal region [9,32,33]. In clinical settings, similar mechanics are known to cause lens dislocation, zonular rupture, and even limbal or globe rupture during severe blunt trauma [34,35,36]. Unlike the posterior pole that experienced alternating compressive and tensile stress, the equator was subjected to radial stretching driven by zonular tension and lens displacement. Correspondingly, the histology of retinoschisis and tear at the equatorial region matches the zones under tensile loading.

Given that traumatic vitreoretinal injuries are typically diagnosed through patient history and dilated fundus examination, early recognition of retinal injury could be critical for timely intervention [37]. However, diagnosis could be delayed due to late symptom onset, limited visualization of the retinal periphery, and concerns about exacerbating anterior segment injury with pharmacologic dilation [38]. The concordance between our FEA and experimental model findings supports the value of controlled trauma models in elucidating these biomechanical injury pathways, with the goal of improving early detection and prevention of trauma-induced retinal detachment. Furthermore, these biomechanical insights can inform the design of protective equipment. For instance, safety goggles could be engineered not only to prevent penetration but also to absorb and redistribute impact energy in a way that dampens the damaging oscillatory intraocular pressure and stress waves identified in this and other studies [39,40].

We acknowledge several limitations that provide avenues for future research. First, both the FEA and experiment utilized the rigid plate to support the eyeball, which somewhat simplifies the real-world injury scenario. In clinical settings, the surrounding retrobulbar fat and orbital muscle may deform with the eye, which may alter the load transfer and energy dissipation during blunt trauma [41,42]. The absence of those tissues in our model may elevate the peak bulging of the globe and lens motion, which therefore exaggerates peak compressive and tensile stress, particularly at the posterior pole and equatorial region. To produce the comparable level of ocular tissue damage under the simplified setup, we used a relatively low height to impact the eye to reduce energy. The reported stress value should be interpreted as an upper-bound estimate rather than a direct in vivo stress evaluation. Nevertheless, our study strengthens the understanding of the spatial and temporal pattern of retinal injury following blunt ocular trauma. Second, the murine eye, while excellent for controlled study, has anatomical differences from the human eye (for example, a larger lens-to-eye volume ratio) [43]. The next critical step is translating this framework into a high-fidelity human-eye model that includes orbital anatomy. Moreover, our FEM model does not fully capture the viscoelastic behavior due to the simplification in material properties for the immediate blunt trauma scenario where the large, nonlinear deformation is dominant [44]. Finally, our model did not define tissue injury based on stress thresholds. Integrating injury criteria based on strain or a combination of stress–strain metrics and validating them against a broader range of histological outcomes (including different grades of hemorrhage and photoreceptor damage) would enhance the model’s predictive power for clinical outcomes.

## 5. Conclusions

This study combines finite element analysis with an in vitro weight-drop test on the murine model to elucidate the biomechanical pathways underlying traumatic retinal detachment. The simulations captured the full sequence of globe deformation during blunt impact, revealing the pronounced compressive–tensile stress oscillation at the posterior pole, compressive stress at the peripheral region, and minimal compressive–tensile oscillation at the equatorial region. These locations under the tensile stress overlapped with histological evidence of retinal detachment and structural disruption, indicating the key role of tensile stress in causing retinal injury during blunt trauma. Together, these findings reveal how the blunt ocular trauma produces spatially distinct patterns of retinal injury with region-specific retinal loading. The framework of this study may help improve diagnostic strategies and enhance early identification of trauma-induced retinal detachment.

## Figures and Tables

**Table 1:** Mechanical properties in the eye model.

Structure	Mesh Number	Material Model	Material Parameters
Cornea [21]	15,265	Neo-Hookean hyperelastic	µ = 0.079 MPa, D_1_ = 1 × 10^−7^ MPa
Sclera [22]	144,570	Yeoh’s 3rd hyperelastic	C_10_ = 0.91 MPa, C_20_ = 19.02 MPa; C_30_ = −64.73 MPa
Lens [22]	29,843	Neo-Hookean hyperelastic	µ = 2.29 MPa, D_1_ = 1 × 10^−7^ MPa
Retina [23]	244,564	Neo-Hookean hyperelastic	µ = 0.005 MPa, D_1_ = 1 × 10^−7^ MPa
Zonules [24]	17,559	Linear elastic	E = 0.35 MPa, ν = 0.47
Aqueous [12]	Eulerian	Shock EOS linear	C_1_ = 1.64 × 106 mm∙s^−1^, S_1_ = 1.92
Vitreous [12]	Eulerian	Shock EOS linear	C_1_ = 1.64 × 106 mm∙s^−1^, S_1_ = 1.92

µ, initial shear modulus; D_1_, incompressibility parameter; C_10_, C_20_ and C_30_, material constants for Yeoh’s 3rd; E, elastic modulus; ν, Poisson’s ratio; C_1_, speed of sound through the material; S_1_, the coefficient related to the speed of the shocked material.

**Table 2:** Comparison between FEA-predicted stress distribution and in vitro histological findings related to detachment across different retinal regions.

Region	FEA	In Vitro Experiment
Posterior pole	Compressive stress during impact; tensile stress during retraction.	Evidence of retinoschisis or detachment observed in photoreceptor layers
Peripheral	Compressive stress during impact and retraction	Minimal structural disruption; intact retinal layers
Equatorial	Radial stress variation during impact and retraction	Detachment observed in pigment epithelium and photoreceptor layers.

## Data Availability

Data available on request from the corresponding authors.

## Supplementary Materials

The following supporting information can be downloaded at: https://www.mdpi.com/article/10.3390/bioengineering13040431/s1, Supplementary S1: Histological study of impacted eyeballs.

## Abbreviations

The following abbreviations are used in this manuscript:

FEA	Finite Element Analysis
H&E	Hematoxylin and Eosin
OCT	Optical Coherence Tomography
RPE	Retinal Pigment Epithelium
σ_r_,σ_θ_,σ_φ_	Radial, Circumferential and Azimuthal Stress
TRD	Traumatic Retinal Detachment

## References

[1] Thylefors B. (1992). Epidemiological Patterns of Ocular Trauma. Aust. N. Z. J. Ophthalmol..

[2] Li C., Fu Y., Liu S., Yu H., Yang X., Zhang M., Liu L. (2023). The Global Incidence and Disability of Eye Injury: An Analysis from the Global Burden of Disease Study 2019. eClinicalMedicine.

[3] Iftikhar M., Latif A., Farid U.Z., Usmani B., Canner J.K., Shah S.M.A. (2019). Changes in the Incidence of Eye Trauma Hospitalizations in the United States from 2001 Through 2014. JAMA Ophthalmol..

[4] Kuhn F., Morris R., Witherspoon C.D., Mann L. (2006). Epidemiology of Blinding Trauma in the United States Eye Injury Registry. Ophthalmic Epidemiol..

[5] Rohowetz L., Fan J., Flynn H. (2025). Vitreoretinal Injury Associated with Sports Ball Ocular Trauma. Clin. Ophthalmol..

[6] Sebag J. (1992). Anatomy and Pathology of the Vitreo-Retinal Interface. Eye.

[7] Ghazi N.G., Green W.R. (2002). Pathology and Pathogenesis of Retinal Detachment. Eye.

[8] Blanch R.J., Ahmed Z., Sik A., Snead D.R.J., Good P.A., O’Neill J., Berry M., Scott R.A.H., Logan A. (2012). Neuroretinal Cell Death in a Murine Model of Closed Globe Injury: Pathological and Functional Characterization. Investig. Ophthalmol. Vis. Sci..

[9] Sponsel W.E., Gray W., Scribbick F.W., Stern A.R., Weiss C.E., Groth S.L., Walker J.D. (2011). Blunt Eye Trauma: Empirical Histopathologic Paintball Impact Thresholds in Fresh Mounted Porcine Eyes. Investig. Ophthalmol. Vis. Sci..

[10] Chen D., Liu X., Sun X., Liu Y., Geng X., Huo H., Tang M., Tang Z., Dong Y., Wang J. (2023). Experimental Evidence to Understand Mechanical Causes of Retinal Detachment Following Blunt Trauma. Exp. Eye Res..

[11] Beckmann L., Cai Z., Cole J., Miller D.A., Liu M., Grannonico M., Zhang X., Ryu H.J., Netland P.A., Liu X. (2021). In Vivo Imaging of the Inner Retinal Layer Structure in Mice after Eye-Opening Using Visible-Light Optical Coherence Tomography. Exp. Eye Res..

[12] Liu X., Wang L., Wang C., Sun G., Liu S., Fan Y. (2013). Mechanism of Traumatic Retinal Detachment in Blunt Impact: A Finite Element Study. J. Biomech..

[13] Rossi T., Boccassini B., Esposito L., Iossa M., Ruggiero A., Tamburrelli C., Bonora N. (2011). The Pathogenesis of Retinal Damage in Blunt Eye Trauma: Finite Element Modeling. Investig. Ophthalmol. Vis. Sci..

[14] Hong J.D., Colmenarez J.A., Choi E.H., Suh A., Suh A., Lam M., Hoskin A., Minckler D.S., Lin K.Y., Shahraki K. (2025). Finite Element Analysis of Mechanical Ocular Sequelae from Badminton Shuttlecock Projectile Impact. Ophthalmol. Sci..

[15] Rydz C., Colmenarez J.A., Shahraki K., Dong P., Gu L., Suh D.W. (2025). Finite Element Analysis of Ocular Impact Forces and Potential Complications in Pickleball-Related Eye Injuries. Bioengineering.

[16] Mohd Rasidin A.H., Muhammad-Ikmal M.K., Raja Omar R.N., Yaakub A., Ahmad Tajudin L.S. (2022). Clinical Audit on Badminton-Related Ocular Injuries in a Tertiary Hospital in Malaysia. Cureus.

[17] Rangarajan N., Kamalakkannan S.B., Hasija V., Shams T., Jenny C., Serbanescu I., Ho J., Rusinek M., Levin A.V. (2009). Finite Element Model of Ocular Injury in Abusive Head Trauma. J. Am. Assoc. Pediatr. Ophthalmol. Strabismus.

[18] Gray W., Sponsel W.E., Scribbick F.W., Stern A.R., Weiss C.E., Groth S.L., Walker J.D. (2011). Numerical Modeling of Paintball Impact Ocular Trauma: Identification of Progressive Injury Mechanisms. Investig. Ophthalmol. Vis. Sci..

[19] Chen D., Sun X., Wu Y., Tang M., Wang J., Qiao X., Zhu Y., Zhang Z., Du X., Guo J. (2024). A Finite Element Model of the Eye Matched with in Vitro Experiments for the Prediction of Traumatic Retinal Detachment. Theor. Appl. Mech. Lett..

[20] Pang J., Le L., Zhou Y., Tu R., Hou Q., Tsuchiya D., Thomas N., Wang Y., Yu Z., Alexander R. (2021). NOTCH Signaling Controls Ciliary Body Morphogenesis and Secretion by Directly Regulating Nectin Protein Expression. Cell Rep..

[21] Zhai Y., Wang J., Mendoza V.O., Ye M., Shahraki K., Suh D.W., Minckler D.S., Karpova T., Nunes K., Dong P. (2025). Spatial Relationship between Histological Staining Intensity and Corneal Stiffness Variations: Insights from AFM Indentation in Infant African Green Monkeys. J. Mech. Behav. Biomed. Mater..

[22] Colmenarez J.A., Zhai Y., Mendoza V.O., Dong P., Nunes K., Suh D., Gu L. (2024). Damage-Induced Softening of the Sclera: A Pseudo-Elastic Modeling Approach. J. Eng. Sci. Med. Diagn. Ther..

[23] Franze K., Francke M., Günter K., Christ A.F., Körber N., Reichenbach A., Guck J. (2011). Spatial Mapping of the Mechanical Properties of the Living Retina Using Scanning Force Microscopy. Soft Matter.

[24] Ariza-Gracia M.Á., Wu W., Calvo B., Malvè M., Büchler P., Rodriguez Matas J.F. (2018). Fluid–Structure Simulation of a General Non-Contact Tonometry. A Required Complexity?. Comput. Methods Appl. Mech. Eng..

[25] Aung O., Rossi P.J., Dyer M.R., Stellpflug A., Zhai Y., Kenneth A., Wang X., Chang J., Chen Y., Tefft B. (2024). Biofabrication of Small Vascular Graft with Acellular Human Amniotic Membrane: A Proof-of-Concept Study in Pig. Biofabrication.

[26] Delori F., Pomerantzeff O., Cox M.S. (1969). Deformation of the Globe under High-Speed Impact: Its Relation to Contusion Injuries. Investig. Ophthalmol. Vis. Sci..

[27] Chauhan K., Dave V.P., De Ribot F.M., Agrawal R., Sallam A.B., Andayani G., Chang C.-J., Hsiao C.-H., Bastion M.-L.C., Hattenbach L.-O. (2025). Traumatic Retinal Detachment: A Contemporary Update. Surv. Ophthalmol..

[28] Jia X., Yu J., Liao S.-H., Duan X.-C. (2016). Biomechanics of the Sclera and Effects on Intraocular Pressure. Int. J. Ophthalmol..

[29] Ahn S.J., Woo S.J., Park K.H., Lee B.R. (2017). Retinal Pigment Epithelium Sequelae Caused by Blunt Ocular Trauma: Incidence, Visual Outcome, and Associated Factors. Sci. Rep..

[30] Morad Y., Wygnansky-Jaffe T., Levin A.V. (2010). Retinal Haemorrhage in Abusive Head Trauma. Clin. Exper Ophthalmol..

[31] Faghihi H., Ghassemi F., Falavarjani K.G., Saeedi Anari G., Safizadeh M., Shahraki K. (2014). Spontaneous Closure of Traumatic Macular Holes. Can. J. Ophthalmol..

[32] Zigiotti G.L., Cavarretta S., Morara M., Nam S.M., Ranno S., Pichi F., Lembo A., Lupo S., Nucci P., Meduri A. (2012). Standard Enucleation with Aluminium Oxide Implant (Bioceramic) Covered with Patient’s Sclera. Sci. World J..

[33] Frisina R., Besozzi G., Gius I., Greggio A., De Salvo G., Meduri A. (2022). Pole to Pole Surgery in Ocular Trauma: Standardizing Surgical Steps. Ophthalmol. Ther..

[34] Bhatia K., Sharma R. (2013). Eye Emergencies. Emergency Medicine.

[35] Wang S., Li F., Jin S., Zhang Y., Yang N., Zhao J. (2023). Biomechanics of Open-Globe Injury: A Review. BioMed. Eng. OnLine.

[36] Stitzel J.D., Duma S.M., Cormier J.M., Herring I.P. (2002). A Nonlinear Finite Element Model of the Eye with Experimental Validation for the Prediction of Globe Rupture. Stapp. Car. Crash J..

[37] Lam M.R., Colmenarez J.A., Dong P., Gu L., Suh D.W. (2024). Vascular Insult in Neonatal Retinal Hemorrhage: Computational Analysis of a Fundus-Segmented Blood Vessel Network. Sci. Rep..

[38] Hoogewoud F., Chronopoulos A., Varga Z., Souteyrand G., Thumann G., Schutz J.S. (2016). Traumatic Retinal Detachment—The Difficulty and Importance of Correct Diagnosis. Surv. Ophthalmol..

[39] Mishra A., Bhirud A., Agrawal M., Tripathi A., Baranwal V.K., Kapoor G. (2024). A Prospective Study to Evaluate the Effectiveness of Preventive Aspects in Relation to Sports Related Ocular Injuries. Int. Ophthalmol..

[40] Mazarelo J.F.D., Winter S.L., Fong D.T.P. (2024). A Systematic Review on the Effectiveness of Eyewear in Reducing the Incidence and Severity of Eye Injuries in Racket Sports. Physician Sportsmed..

[41] Cohen L.M., Habib L.A., Yoon M.K. (2020). Post-Traumatic Enophthalmos Secondary to Orbital Fat Atrophy: A Volumetric Analysis. Orbit.

[42] Jafari S., Hollister J., Kavehpour P., Demer J.L. (2024). Shear Viscoelastic Properties of Human Orbital Fat. J. Biomech..

[43] Evans L.P., Newell E.A., Mahajan M., Tsang S.H., Ferguson P.J., Mahoney J., Hue C.D., Vogel E.W., Morrison B., Arancio O. (2018). Acute Vitreoretinal Trauma and Inflammation after Traumatic Brain Injury in Mice. Ann. Clin. Transl. Neurol..

[44] Karimi A., Razaghi R., Sera T., Kudo S. (2019). A Combination of the Finite Element Analysis and Experimental Indentation via the Cornea. J. Mech. Behav. Biomed. Mater..

