# Supplementary material for: Spatiotemporal Mapping of Biomechanical Stress Predicts Region-Specific Retinal Injury in a Murine Model of Blunt Ocular Trauma
Source: Bioengineering (Basel). 2026 Apr 7;13(4):431. doi: 10.3390/bioengineering13040431 (PMC13112936; doi:10.3390/bioengineering13040431)

Histological study on impacted eyeballs. Black arrows mark the sites of retinal detachment or stretching. All images orient eyes with the cornea facing up

Eyeball I:

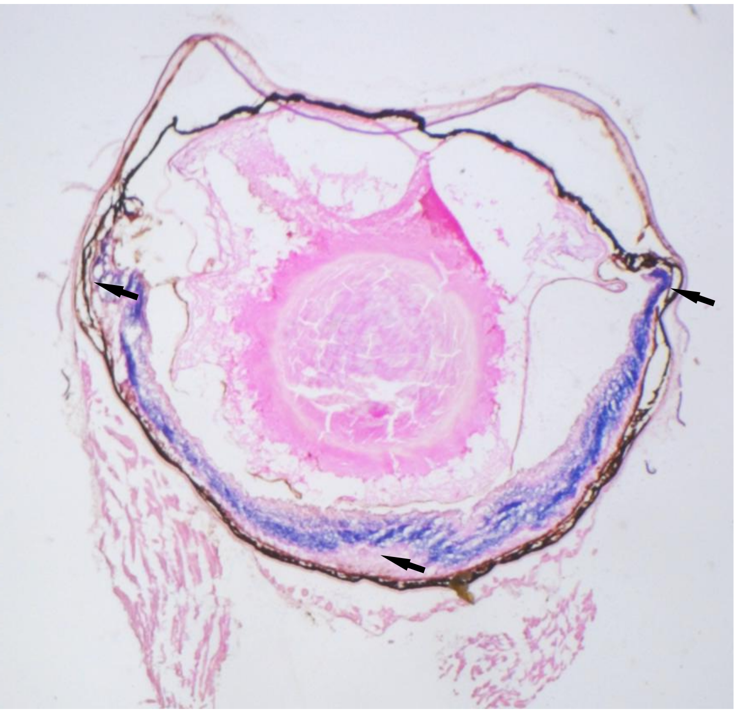

Eyeball II:

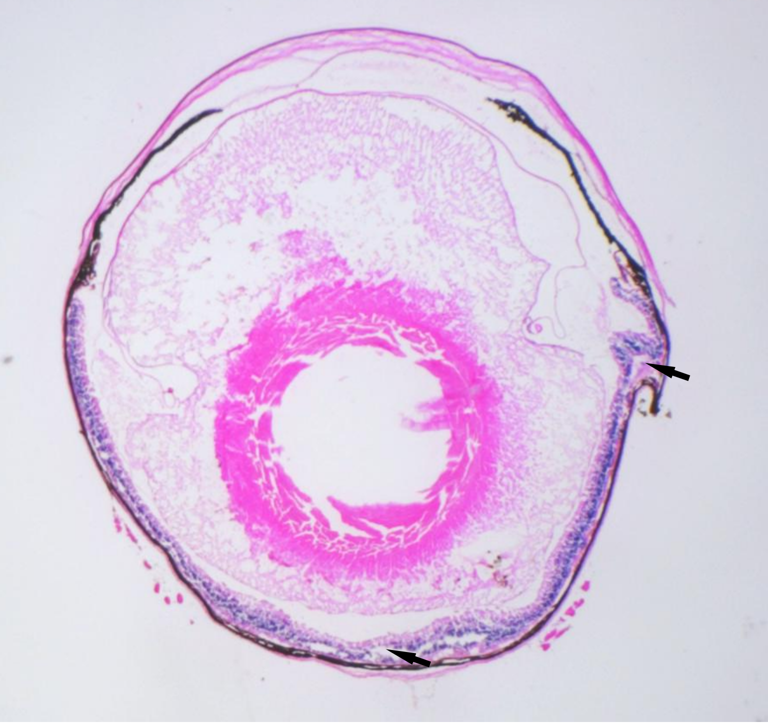

Eyeball III:

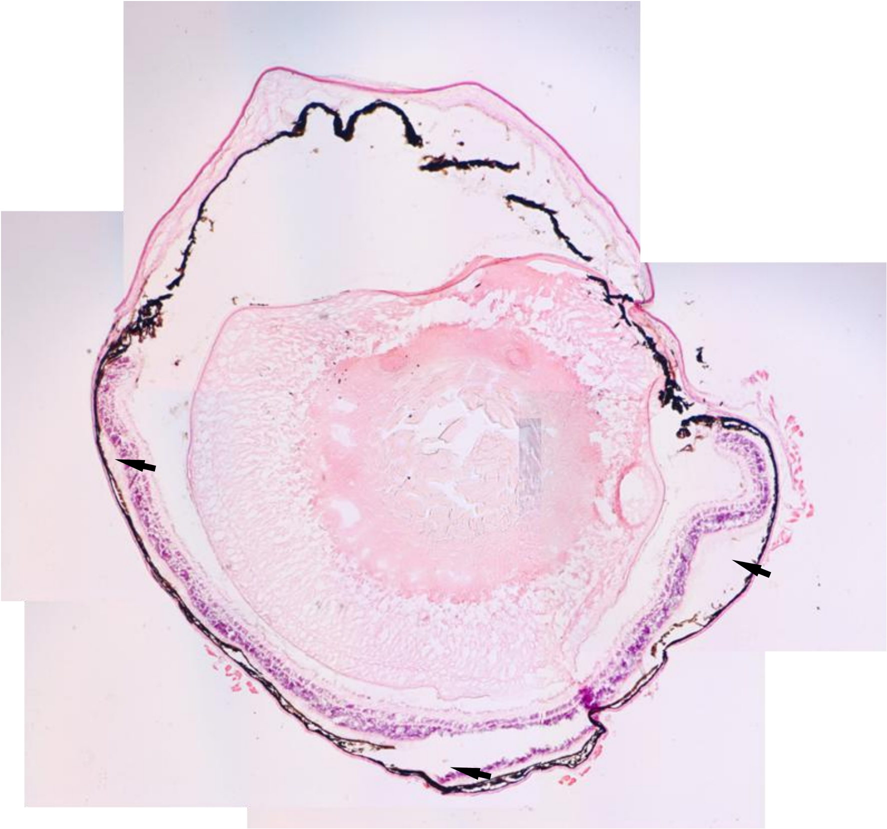

Retinal histology on posterior pole, peripheral and equatorial region of the eye compared with the control group

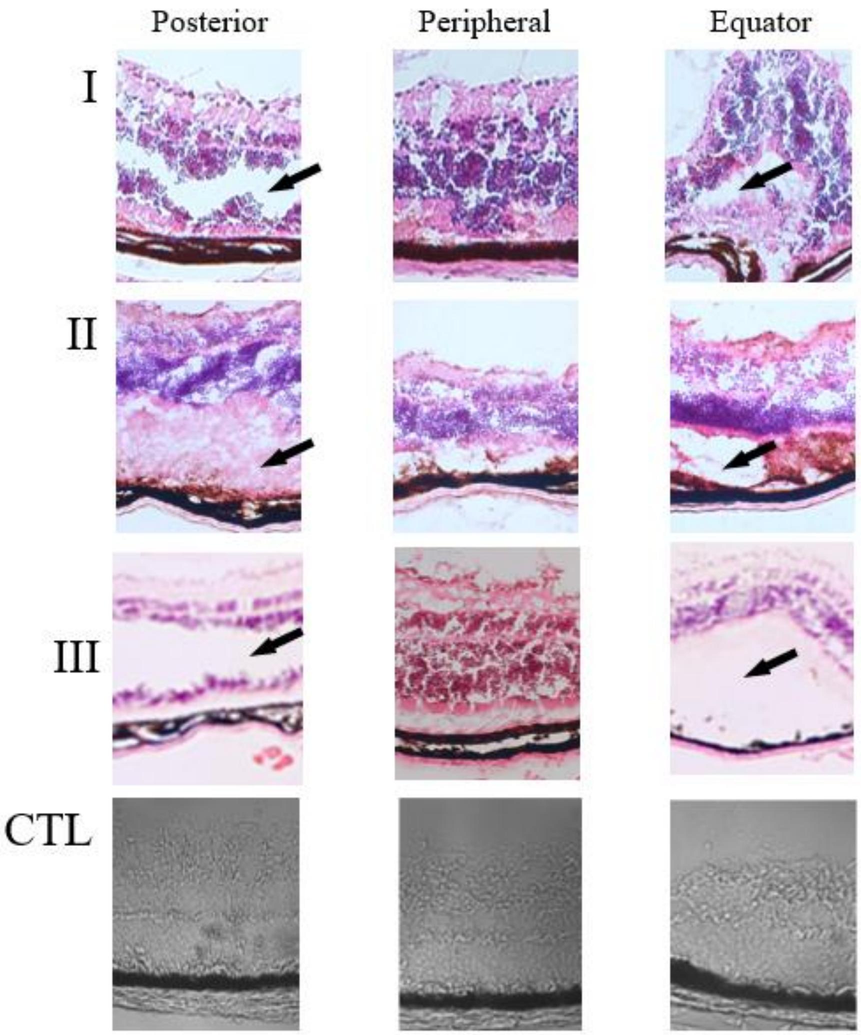

Supplement: Supplementary file 1 [file bioengineering-13-00431-s001.zip › bioengineering-4175834-supplementary.pdf]
